# Evaluating a New Digital App–Based Program for Heart Health: Feasibility and Acceptability Pilot Study

**DOI:** 10.2196/50446

**Published:** 2024-05-24

**Authors:** Kimberly G Lockwood, Priya R Kulkarni, Jason Paruthi, Lauren S Buch, Mathieu Chaffard, Eva C Schitter, OraLee H Branch, Sarah A Graham

**Affiliations:** 1 Lark Health Mountain View, CA United States; 2 Roche Information Solutions Santa Clara, CA United States; 3 Anara Health Los Angeles, CA United States

**Keywords:** digital health, cardiovascular disease, artificial intelligence, AI, acceptability and feasibility, pilot study, lifestyle coaching, mobile phone

## Abstract

**Background:**

Cardiovascular disease (CVD) is the leading cause of death in the United States, affecting a significant proportion of adults. Digital health lifestyle change programs have emerged as a promising method of CVD prevention, offering benefits such as on-demand support, lower cost, and increased scalability. Prior research has shown the effectiveness of digital health interventions in reducing negative CVD outcomes. This pilot study focuses on the Lark Heart Health program, a fully digital artificial intelligence (AI)–powered smartphone app, providing synchronous CVD risk counseling, educational content, and personalized coaching.

**Objective:**

This pilot study evaluated the feasibility and acceptability of a fully digital AI-powered lifestyle change program called Lark Heart Health. Primary analyses assessed (1) participant satisfaction, (2) engagement with the program, and (3) the submission of health screeners. Secondary analyses were conducted to evaluate weight loss outcomes, given that a major focus of the Heart Health program is weight management.

**Methods:**

This study enrolled 509 participants in the 90-day real-world single-arm pilot study of the Heart Health app. Participants engaged with the app by participating in coaching conversations, logging meals, tracking weight, and completing educational lessons. The study outcomes included participant satisfaction, app engagement, the completion of screeners, and weight loss.

**Results:**

On average, Heart Health study participants were aged 60.9 (SD 10.3; range 40-75) years, with average BMI indicating class I obesity. Of the 509 participants, 489 (96.1%) stayed enrolled until the end of the study (dropout rate: 3.9%). Study retention, based on providing a weight measurement during month 3, was 80% (407/509; 95% CI 76.2%-83.4%). Participant satisfaction scores indicated high satisfaction with the overall app experience, with an average score of ≥4 out of 5 for all satisfaction indicators. Participants also showed high engagement with the app, with 83.4% (408/489; 95% CI 80.1%-86.7%) of the sample engaging in ≥5 coaching conversations in month 3. The results indicated that participants were successfully able to submit health screeners within the app, with 90% (440/489; 95% CI 87%-92.5%) submitting all 3 screeners measured in the study. Finally, secondary analyses showed that participants lost weight during the program, with analyses showing an average weight nadir of 3.8% (SD 2.9%; 95% CI 3.5%-4.1%).

**Conclusions:**

The study results indicate that participants in this study were satisfied with their experience using the Heart Health app, highly engaged with the app features, and willing and able to complete health screening surveys in the app. These acceptability and feasibility results provide a key first step in the process of evidence generation for a new AI-powered digital program for heart health. Future work can expand these results to test outcomes with a commercial version of the Heart Health app in a diverse real-world sample.

## Introduction

### Background

Cardiovascular disease (CVD) is the leading cause of death for adults in the United States [1], and 49.2% of adults (126.9 million) were living with some form of CVD in 2018 [2]. The primary type of CVD that drives CVD-related morbidity and mortality is atherosclerotic CVD (ASCVD), which is characterized by buildup of plaque in arteries and includes conditions such as coronary heart disease, cerebrovascular disease, peripheral artery disease, and aortic atherosclerotic disease [3]. Despite effective evidence-based strategies for preventing or managing ASCVD, millions of Americans have risk factors that place them at increased risk for having a cardiovascular event [1]. Thus, the prevention and management of CVD is a top public health priority.

The primary method of preventing CVD is promoting a healthy lifestyle throughout life. The American College of Cardiology (ACC) and American Heart Association (AHA) guidelines on the primary prevention of CVD [3] recommend that all adults should maintain a healthy weight, consume a healthy diet, engage in regular physical activity, avoid tobacco use, and practice good sleep hygiene. These lifestyle habits are also recommended for the secondary prevention of CVD. The ACC and AHA guidelines also recommend that adults who are aged 40 to 75 years and are being evaluated for CVD risk should undergo a 10-year ASCVD risk estimation at the initial evaluation and at each subsequent follow-up appointment.

Despite these guidelines, maintaining a healthy lifestyle is difficult for many adults [4]. As such, a key method of CVD prevention and management is participation in behavioral interventions focused on lifestyle modifications. The US Preventive Services Task Force advises that behavioral interventions focused on diet and physical activity provide cardiovascular benefits to adults both with and without known CVD risk [5-7]. Interventions and lifestyle change programs can be focused on primary prevention of CVD and secondary prevention after a cardiac event and can be delivered by a variety of modalities (eg, in person, by telephone, and digitally) but have traditionally been delivered in person [7,8].

Although in-person programs can be very effective for increasing healthy behaviors [5,6], they also present many challenges. Such programs involve significant human-to-human contact (eg, nurses and coaches), making them expensive to implement. In addition, requiring in-person attendance makes it difficult or inconvenient for some individuals to attend [9]. These barriers to implementation and participation make it challenging to scale lifestyle interventions to large populations. Given the large number of adults in the United States at risk for CVD, there is a clear need for scalable and accessible lifestyle change programs for preventing CVD.

There is a growing number of digital lifestyle change programs for primary and secondary CVD prevention [10-12] that can be delivered through a variety of digital modalities (eg, SMS text messaging, smartphone apps, and web-based tools). Digital health programs can avoid many of the challenges posed by in-person programs, offering benefits such as on-demand support, access after standard business hours, lower cost, and increased scalability to larger populations [8]. Furthermore, digital health programs for primary and secondary CVD prevention have been shown to improve individual behaviors and show promise in delivering care that is accessible, cost-effective, and patient focused [13].

### Digital Health Solutions for CVD Prevention

There has been prior research on digital health interventions for the prevention of CVD [13-15]. A meta-analysis by Widmer et al [15] of digital health interventions for both the primary and secondary prevention of CVD summarized many of these findings in an assessment of 24,054 participants across 51 studies. This meta-analysis found that digital health interventions significantly reduced negative CVD outcomes, such as CVD events, hospitalizations, and all-cause mortality. Widmer et al [15] also observed reductions in risk factors, including weight, BMI, and Framingham risk score, compared to usual care. A more recent analysis that focused on health behaviors also indicated that digital interventions can improve physical activity, diet quality, and medication adherence [16]. Overall, these studies show a net benefit of digital health interventions on CVD outcomes and risk factors.

As demonstrated by these prior studies, there is evidence for the benefits of digital health interventions for the primary and secondary prevention of CVD. However, there has been considerable variation in the delivery mode of available digital solutions, namely in the involvement of humans as coaches or health care providers. A common feature across most digital health programs is the inclusion of human coaching or care provision delivered via telephone calls, SMS text messaging, or email [17]. Moreover, a recent review of the literature indicated that very few (4/31, 13%) cardiovascular health–related digital health interventions are fully automated, requiring no specific personnel [11]. Human interaction elements in digital health, similar to in-person programs, require significant resources, limiting their scalability [18]. As a result, this pilot study examined a fully digital solution (ie, no human-to-human coaching) for primary and secondary CVD prevention called Lark Heart Health.

### Heart Health Program and Pilot Study

The primary purpose of the Heart Health program is to help participants make and maintain meaningful evidence-based healthy lifestyle changes, learn about their CVD risk factors, and acquire appropriate self-management skills. The Lark Heart Health program is an artificial intelligence (AI)–driven mobile coaching solution that provides synchronous CVD risk counseling anytime, anywhere. The Heart Health program is for primary prevention in individuals without a major ASCVD event or secondary prevention for those in a stable condition after an ASCVD event that occurred ≥6 months prior. Through an ASCVD Risk Estimator survey, evidence-based educational curriculum, and real-time personalized coaching, the Lark Heart Health program provides members with the tools they need to make meaningful lifestyle changes that can help them better prevent and manage ASCVD and coronary artery disease through heart health–specific digital nutrition coaching; medication adherence counseling; and personalized guidance on weight management, activity, stress, and sleep. In addition, one of the goals of the Heart Health program is to provide a user-friendly platform for participants to complete physical and mental health screening surveys that can be time consuming and burdensome for both patients and providers in primary care settings [5,19]. This is particularly important because the completion of health screeners is associated with improved patient outcomes and clinical care [20].

Experts designed the Heart Health program in accordance with guidelines from the AHA; the ACC; and the National Heart, Lung, and Blood Institute. The American Diabetes Association 2020-2025 Dietary Guidelines for Americans, the American College of Sports Medicine, and the American Academy of Sleep Medicine informed the additional nutrition, activity, and sleep recommendations. The development of the Heart Health program was a collaboration between Lark Health and Roche Diagnostics and fits within the World Heart Federation Roadmap for Digital Health in Cardiology [21]. Full details on Lark’s other digital programs have been previously reported [22,23].

Coaching in the Lark Heart Health program is completely powered by AI. The fully digital interface enables maximum program scalability and the advantage of being accessible to participants 24/7 with synchronous coaching, feedback, and encouragement delivered on demand. Feasibility and acceptability studies are a key component of evidence generation for new programs of any kind and provide a crucial preliminary step before conducting randomized controlled trials and commercialization. Similar published studies provide examples of the overall scope, design, and execution of digital programs for cardiovascular health, CVD, and other chronic diseases [24-26]; for example, Lunde et al [24] conducted a feasibility study of an app for cardiac rehabilitation, setting predefined criteria for success on participant satisfaction, recruitment rates, and app adherence to help predict possible pitfalls before conducting a large randomized controlled trial. Despite these inherent advantages, there is little evidence supporting the feasibility and user acceptability of fully digital programs for cardiovascular health. Thus, the goal of this pilot study was to provide preliminary evidence that participants are willing and able to participate in the Heart Health program.

### Objectives

This is a real-world single-arm 3-month pilot study of the feasibility and acceptability of the Heart Health program. The primary objectives and corresponding success criteria determined a priori are outlined in the following subsections.

#### Objective 1

Assess participant satisfaction with the Heart Health program (primary indicator of success: achieve a participant satisfaction average rating of ≥4 out of 5 on participant satisfaction surveys).

#### Objective 2

Measure participant engagement with the Heart Health program through the frequency of AI coaching (primary indicator of success: assess the number of coaching conversations per month and categorize them as *highly engaged,* ≥5 coaching conversations per month; *moderately engaged,* 2-4 conversations per month; and *minimally engaged*, ≤1 conversation per month).

#### Objective 3

Track the submission of health screeners within the Heart Health app to demonstrate that we can identify moments or events that could be used to improve patient care (primary indicator of success: submission of 3 surveys by each member; 3 completed surveys=*excellent*, 2 completed surveys=*good*, and 1 completed survey=*minimum improvement over usual care*; this scale was based on literature indicating that there are major barriers to implementing even basic health history screeners in primary care settings [19,20,27]).

#### Secondary Objective

In addition to the primary objectives, we also conducted secondary analyses focused on weight loss, given that a primary focus of the Heart Health program is weight management.

### Additional Results Reported Elsewhere

As part of the Heart Health pilot study, we also evaluated the predictors of recruitment and enrollment as well as changes in cardiac self-efficacy; the results from these analyses are reported elsewhere [28] because they do not focus on the acceptability and feasibility component of the study.

## Methods

### Pilot Study Design

#### Overview

This pilot study was a real-world single-arm study of the digital health app–based program Heart Health that provides fully digital AI-powered health coaching, behavior tracking, and health screeners. The active study period was 3 months (90 d) in duration for each enrolled participant. The data presented here include participants who initiated the study between March 31 and September 15, 2022, using version 5.2.6 of the Lark app. The target sample size for initial enrollment into this study was 500 to 600; the rationale for this enrollment goal is described in Multimedia Appendix 1 [22,29,30].

#### Study Population

To be eligible for the Heart Health study, potential participants had to meet the following eligibility parameters: aged 40 to 75 years, BMI between 25 kg/m^2^ and 50 kg/m^2^ (indicating overweight or obesity but excluding the most extreme cases of obesity), and English speaking. Potential participants were ineligible if they met any of the following criteria: critically serious uncontrolled health conditions that had been active in the last 6 months, pregnancy or plans to become pregnant within the next 6 months, recent history of a medical professional telling them not to participate in a healthy lifestyle program, a medical reason preventing them from performing 10 minutes of moderate physical exercise, and not having a smartphone with an internet connection. As the Heart Health program focused heavily on improving health behaviors (eg, diet and exercise), the study also excluded individuals who reported at baseline that they regularly engage in strenuous physical activity in their leisure time as well as individuals who reported only healthy dietary behaviors. If needed, study personnel provided potential participants with telephone-based assistance in downloading the Lark smartphone app.

An analytic size of approximately 500 is recommended to produce statistics that are nearly representative of the true values in the targeted population [31]. As such, we considered a sample size between 500 and 600 at initial enrollment to be satisfactory for assessing the primary study goals. This planned sample size at initial enrollment was larger than the sample sizes in most published digital health interventions for the prevention of CVD [15].

**Figure 1:**
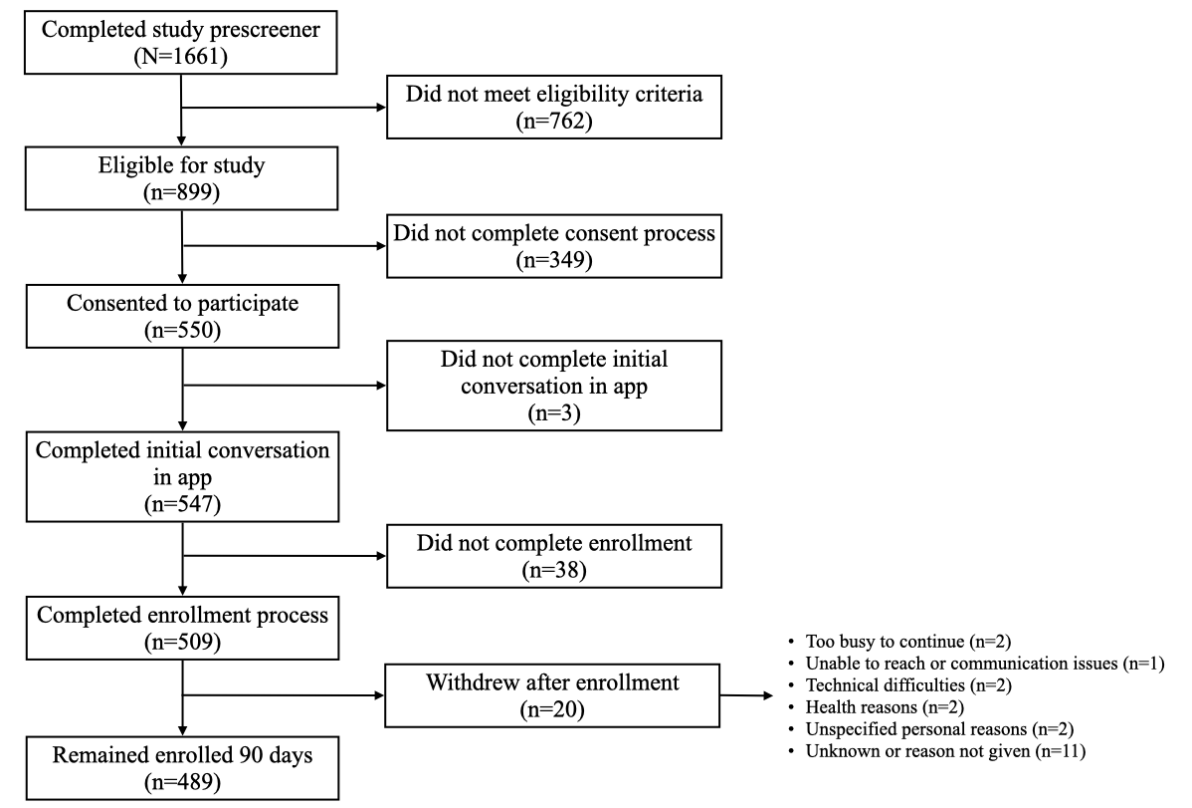
Recruitment and enrollment flow for the Heart Health study.

The recruitment and enrollment flow for the Heart Health study is shown in Figure 1, starting with the number of potential participants who completed the study prescreener. After consenting to participate in the study, participants completed the enrollment process by downloading the app, completing study onboarding in an initial conversation in the app, and providing an initial weight measurement. Per study protocol, participants completing these steps were enrollees. The dropout rate for the study was 3.9%, with 20 of the 509 participants withdrawing after enrollment.

#### Study Phases

Each participant proceeded through defined study phases: recruitment, prescreening, electronic consent, enrollment, and active participation. For recruitment, Lark partnered with a health provider in California to recruit participants from its patient population. Study personnel sent potentially eligible participants marketing emails and SMS text messages as well as printed mailers. In addition to participant recruitment via the health provider, we also recruited additional study participants using a variety of web recruitment methods. The recruitment materials provided a brief description of the program, describing the program as a way to improve heart health using digital health coaching that encourages healthier eating and becoming more active. Individuals interested in participating in the study received a web link to the study recruitment site to find out more information and complete the study prescreening process.

Participants who proceeded from recruitment to prescreening completed a survey that assessed their general suitability for the study based on the safety and appropriateness of the program coaching and content. Participants who passed the prescreening survey had the opportunity to complete the electronic consent form to initiate study participation.

The active participation phase of the study was 90 days in duration, during which participants received coaching on heart health and lifestyle modification. Participants could engage with the Heart Health program by (1) completing educational lessons (up to 12 lessons over 90 days), (2) engaging in coaching conversations with Lark’s conversational AI-powered digital coach, (3) logging meals in the app, and (4) tracking their weight using a digital smart scale that automatically synced with the app. Additional details on the Heart Health program and study design are provided in Multimedia Appendix 1.

### Ethical Considerations

All participants provided informed consent to participate in the study through an electronic consent form. The pilot study received approval from the Advarra Institutional Review Board (Pro00061694). Study personnel instituted appropriate safeguards to prevent any unauthorized use or disclosure of personal health information and implemented administrative, physical, and technical safeguards to protect the confidentiality, integrity, and availability of protected health information. All study data presented in this manuscript are deidentified. Lark is compliant with Health Insurance Portability and Accountability Act (HIPAA) privacy and security rules and all applicable regulations. In addition, Lark is Service Organization Control 2 (SOC 2) and Health Information Trust Alliance (HITRUST) certified.

All study participants received the Heart Health app and a cellular smart scale. There were opportunities for additional incentives throughout the study. At the start of the study, participants could receive a US $50 gift card upon ordering their cellular scale; this incentive was implemented to encourage participant use of the cellular scale for regular weigh-ins for higher data integrity compared to manual entry of weight data. There were two additional opportunities for participants to earn gift card incentives for completing (1) a telephone call regarding customer satisfaction and the usability of the Heart Health app (US $25) and (2) a telephone call about experiences taking surveys in the app (US $100). Additional details on these telephone calls are provided in the *Participant Satisfaction Surveys* subsection. Finally, participants could receive a Fitbit device upon study completion.

### Study Measures

#### Demographic and Health History Measures

We assessed participant characteristics for the study sample using a modified version of the nonlaboratory-based INTERHEART Modifiable Risk Score survey [29,30]. Additional details on this measure are provided in Multimedia Appendix 1. Participants also completed the ASCVD Risk Estimator survey [32,33].

#### Study Retention

We assessed study retention based on the proportion of participants who recorded a weight between days 61 and 90 (month 3) out of the 509 members who enrolled in the study. Study personnel determined this retention metric a priori as a key indicator of whether a participant was still engaging with the Heart Health program.

#### Primary Objective Measures

##### Participant Satisfaction Surveys

Participants completed 2 satisfaction surveys over the course of the study. Importantly, these surveys were specific to this research study and would not typically be part of the commercial Heart Health user experience. As such, the surveys were not built into the digital app experience but instead collected via telephone call by a contract research organization (CRO) contracted by Lark Health for the purposes of this pilot study. Telephone calls were made by call center employees of the CRO who were specifically trained to administer these surveys; the survey calls did not include human coaching that would augment the Lark app experience. This helped ensure that participation satisfaction ratings were not collected by Lark clinical research staff and reduced the impact of these survey assessments on the in-app experience.

To measure participant satisfaction with the overall usability and acceptability of Heart Health, the CRO deployed a 5-item satisfaction survey at the beginning of month 3. We adapted questions on this survey from a recent study on the acceptability of a lifestyle-based heart health risk assessment tool [34]. We also assessed participant satisfaction with the experience of taking the ASCVD Risk Estimator survey in the app using a 3-item satisfaction survey to understand whether participants were capable of, and comfortable with, providing this type of health information without assistance in the Heart Health app. This satisfaction survey also asked participants whether they thought the survey was an appropriate length. The CRO deployed this satisfaction survey at the beginning of month 2. The primary success criterion for satisfaction with both the overall app experience and in-app ASCVD survey experience was a rating of ≥4 out of 5.

##### App Engagement

Participants engage with the Heart Health app primarily through personalized one-on-one interactive coaching conversations between the participant and the Lark AI coach. Participants can log in to the app at any time to engage in a coaching conversation with the coach and receive educational lessons, synchronous feedback, and encouragement. We assessed the number of days that study participants had at least 1 coaching conversation. We assessed the number of days with coaching conversations per month and categorized the coaching volume (the primary indicator of engagement) as follows: ≥5 coaching conversations per month was *highly engaged*, 2 to 4 conversations per month was *moderately engaged*, and ≤1 conversation per month was *minimally engaged*. These engagement thresholds were determined based on the expectations of the health partner for engagement with a commercial version of the program while also accounting for individual differences in engagement and expected engagement drop-off in digital health programs.

Although we did not have specific success criteria for the other metrics of app engagement, we also tracked the number of days participants logged meals, the number of days participants completed weigh-ins (ie, weight measurements), and the number of educational lessons completed. Each of these types of engagement occurs within the context of coaching conversations with the Lark coach. The Heart Health app provides a digital platform for diet tracking via a meal-logging system that uses natural language processing. Participants can log meals and snacks in the app at any time, receive feedback on the nutritional content of their meals, and earn success badges for healthy meals.

The Lark coach encourages participants to weigh themselves using their cellular scale on a weekly basis. The scale automatically syncs with the app so that participants can track their progress. Participants could also enter their weight manually. In our analyses, we assessed the number of days that study participants had at least 1 meal logged and the number of days with a weight measurement in the 90 days since study enrollment.

Finally, we assessed the mean number of educational lessons completed by each participant. A lesson is complete once a participant has completed all content associated with each of the required 7 check-ins. Participants could complete a maximum of 12 lessons over the course of the 90-day study (ie, 1 lesson per week).

##### Screener Submission

The app presented Heart Health members with several screeners throughout their participation in the study. Three key screeners were presented during month 1 of the program: the Patient Health Questionnaire-2 (PHQ-2) depression screener [35], the Medication Adherence Questionnaire (MAQ) [36], and the ASCVD Risk Estimator [32,33]. The PHQ-2 is a 2-item measure that indicates risk for depression. The MAQ has multiple sections, the first of which is a *medication check* that establishes whether the member is taking medications; members who are taking medications proceed to the *adherence* section of the MAQ. Submission values for the MAQ reflect the number of members who completed the medication check questions. Submission values for the ASCVD Risk Estimator reflect the number of members who submitted the survey; it should be noted that participants could submit the ASCVD Risk Estimator without completing all fields necessary to calculate a risk score (eg, participants could skip cholesterol level values because they may not know these values). The primary success criterion for this outcome was the submission of 3 surveys by each participant. Having 3 completed was *excellent*, completing 2 was *good*, and having 1 completed was a *minimum improvement over usual care*.

#### Secondary Objective Measures: Weight Loss

To assess weight loss, we examined weight nadir (ie, peak weight loss) among participants in the Heart Health program who provided at least 2 weight measurements. Participants could complete a weigh-in at any time in the study either by stepping onto their synced cellular scale or manually entering a weight. Outlier detection algorithms ensure the fidelity of all collected weight data (for details, refer to prior Lark publications [22,37]). The weight nadir outcome considered only valid weights and represented each member’s weight loss (first weight - lowest weight) / first weight. We represent the weight nadir as the percentage of initial weight lost.

As the weight nadir could occur at any point in the pilot study, we also assessed the relationship between the day in the study that the weight nadir occurred and the percentage weight loss; a positive correlation indicates that greater percentage weight loss occurred later in the study. We also assessed whether there was a significant relationship between weight nadir and baseline BMI.

### Statistical Analysis

As this pilot study focused on feasibility and acceptability, the results for primary outcomes are largely descriptive in nature, and we evaluated success based on success criteria established a priori*,* described in the *Objectives* subsection. Retention rate, engagement, and screener submission rates were summarized using percentages and 95% CIs for proportions using normal approximation. Participant satisfaction was expressed using means and 95% CIs. For the secondary analyses on weight nadir, we report on the weight nadir across the sample, as described in the previous subsection. In addition we assessed the relationships between (1) weight nadir date and weight loss and (2) baseline BMI and weight loss using Pearson correlations.

## Results

### Participant Characteristics

Table 1 contains basic descriptive statistics of the enrolled study sample. On average, Heart Health study participants were aged 60.9 (SD 10.3; range 40.5-75.9) years and had a BMI indicating class I obesity (mean 32.6, SD 5.9 kg/m^2^; range 24.5-49.6 kg/m^2^). Of the 489 participants, 298 (60.9%) were female; 33 (6.7%) identified as Black, 354 (72.4%) identified as White, and 95 (19.4%) reported belonging to another racial group. An assessment of basic health history indicated that more than half of the sample (286/489, 58.5%) had a history of treatment for high blood pressure, and 19% (93/489) had a history of type 2 diabetes. On the basis of reports of a cardiac event or CVD diagnosis history, 89.8% (439/489) of the participants were in primary prevention, and 10.2% (50/489) were in secondary prevention (ie, history of a cardiac event or CVD diagnosis). ASCVD risk scores for participants in primary prevention who provided complete surveys indicated that 63.8% (118/185) of the participants in this group were borderline risk or higher for developing CVD or having a cardiac event in the next 10 years.

**Table 1:** Baseline characteristics of participants enrolled in the Heart Health study.

Characteristics			Values
Recruitment source (individuals recruited from health partner; n=489), n (%)			326 (66.7)
Age (y; n=489), mean (SD)			60.9 (10.3)
Sex (female; n=489), n (%)			298 (60.9)
**Race (n=489), n (%)**			
	Black	33 (6.7)	
	White	354 (72.4)	
	Other	95 (19.4)	
BMI (kg/m^2^; n=489), mean (SD)			32.6 (5.9)
History of treatment for high blood pressure (n=489), n (%)			286 (58.5)
History of type 2 diabetes (n=489), n (%)			93 (19)
History of cardiac event or CVD^a^ diagnosis (n=489), n (%)			50 (10.2)
**ASCVD^b^ 10-year risk score^c^ (%; n=185), n (%)**			
	Low risk (<5)	67 (36.2)	
	Borderline risk (5-7.4)	15 (8.1)	
	Intermediate risk (7.5-19.9)	70 (37.8)	
	High risk (≥20)	33 (17.8)	
**Tobacco history^d^ (n=489), n (%)**			
	Never smoker	263 (53.8)	
	Former smoker	169 (34.5)	
	Current smoker	43 (8.8)	
**Physical activity in leisure time (n=489), n (%)**			
	Mainly sedentary (eg, sitting, reading, and watching television)	169 (34.6)	
	Mild low-effort exercise (eg, easy walk and yoga)	213 (43.6)	
	Moderate exercise (eg, walking and biking)	107 (21.9)	
**Dietary habits^e^, n (%)**			
	Salty food (daily; n=467)	308 (66)	
	Fried food (≥3 times per week; n=451)	155 (34.4)	
	Meat (≥2 times per day; n=460)	278 (60.4)	
	Fruit (daily; n=463)	239 (51.6)	
	Vegetables (daily; n=459)	324 (70.6)	

^a^CVD: cardiovascular disease.

^b^ASCVD: atherosclerotic cardiovascular disease.

^c^ASCVD 10-year risk scores were only calculated for participants in primary prevention with complete surveys (n=185) and risk categories established by Arnett et al [3].

^d^On the tobacco history questionnaire, 14 (2.9%) of the 489 participants declined to report on tobacco use.

^e^Participants could indicate multiple unhealthy dietary habits. The denominators for dietary habits differ due to a technical issue that affected a subset of participants such that not all dietary habit questions were displayed on the survey.

Regarding health behaviors, a little more than half of the sample (263/489, 53.8%) reported never smoking, while approximately a third of the sample (169/489, 34.6%) were former smokers, and 8.8% (43/489) were current smokers. The majority of the sample (382/489, 78.1%) reported being mainly sedentary or engaging in mild low-effort exercise during their leisure time. All enrolled participants reported at least 1 unhealthy dietary habit, including eating salty food daily, eating fried food ≥3 times per week, eating meat ≥2 times per day, not eating fruit daily, or not eating vegetables daily.

The majority of the participants (481/489, 98.4%) in this sample were obese. Specifically, 36.4% (178/489) of the participants were overweight, 34.4% (168/489) were classified as obese class I, 14.3% (70/489) as obese class II, and 13.3% (65/489) as obese class III. Of the 489 participants, 8 (1.6%) reported a BMI between 24 and 25 kg/m^2^ (ie, normal weight) at baseline; we considered these individuals eligible (ie, BMI ≥25 kg/m^2^) during prescreening because they provided a slightly higher weight on the prescreener survey.

### Study Retention

In month 3, study retention based on providing a weigh-in after day 60 was 80% (407/509; 95% CI 76.2%-83.4%). This month 3 retention rate is comparable to benchmarks from non-AI digital health programs [38].

### Primary Objective 1: Participant Satisfaction

For objective 1, we evaluated participant satisfaction with the Heart Health program. Overall, participants were highly satisfied with the overall app experience (Table 2). On average, participants scored the Lark Heart Health app experience ≥4 (favorable) for each question asked. Members were also highly satisfied with their experience taking the ASCVD survey in the app (Table 2). On average, participants provided high ratings of their comfort with, and understanding of, the ASCVD survey; participants also considered the survey length to be appropriate (422/458, 92.1%, 95% CI 89.3%-94.4% gave it a rating of 3 [*just right*]).

**Table 2:** Participant satisfaction (measured on a scale ranging from 1 to 5) survey results.

Survey items		Values, mean (SD; 95% CI)
**Satisfaction with the overall Lark Heart Health app experience^a^**		
	How interested are you in modifying your lifestyle choices to reduce your cardiovascular risk?	4.7 (0.6; 4.6-4.7)
	How interested are you in using the Lark app to better understand your cardiovascular risk?	4.2 (1.1; 4.1-4.3)
	How useful has the information provided in the app missions [lessons] been?	4.2 (1.0; 4.1-4.3)
	How easy to understand was the information provided in the missions [lessons] in the Lark app?	4.7 (0.6; 4.6-4.7)
	How favorably does using the Lark app compare to how you previously managed your health?	4.2 (1.0; 4.1-4.3)
**Satisfaction with taking the ASCVD^b^ survey in the Lark Heart Health app^c^**		
	How comfortable did you feel taking this survey on your own in the app?	4.8 (0.6; 4.7-4.9)
	How would you rate your understanding of the questions asked in the survey?	4.9 (0.3; 4.9-4.9)

^a^n=432 members completed the app experience satisfaction survey.

^b^ASCVD: atherosclerotic cardiovascular disease.

^c^n=458 members completed the ASCVD survey satisfaction survey.

### Primary Objective 2: App Engagement

For objective 2, we found that participants were highly engaged with coaching in the Heart Health app (Table 3). In month 1, of the 489 participants, 483 (98.8%) met the success criteria for being highly engaged (ie, ≥5 coaching conversations); in month 2, there were 445 (91%) highly engaged participants; and in month 3, there were 408 (83.4%) highly engaged participants. When examining the total number of coaching conversations over the course of the program, participants averaged 129.8 (SD 110.1; median 104.0; 95% CI 120.0-139.6) coaching conversations across the 90-day study period, or 1.4 conversations per day. There was substantial variability in the number of coaching conversations across participants, as indicated by the large SD in total coaching conversations. The median number of coaching conversations was lower for the total number of conversations, indicating that the means may be skewed by extremely engaged users. However, the median values still indicate high engagement.

**Table 3:** Coaching conversations during the Heart Health study by month in the study (n=489).

	≥5 coaching conversations, n (%; 95% CI)	2 to 4 coaching conversations, n (%; 95% CI)	≤1 coaching conversation, n (%; 95% CI)
Month 1	483 (98.8; 97.80-99.85)	6 (1.2; 0.30-2.20)	0 (0; 0-0)
Month 2	445 (91; 88.50-93.50)	26 (5.3; 3.30-7.30)	18 (3.7; 2.00-5.40)
Month 3	408 (83.4; 80.10-86.70)	39 (8; 5.60-10.40)	42 (8.6; 6.10-11.10)

We also tracked the number of days with meals logged, days with weigh-ins, and total number of educational lessons completed across the 90-day study period, all of which occur within coaching conversations with the Lark coach. On average, participants logged meals on 57.0 (SD 30.1; median 68.0; 95% CI 54.3-59.7) days, nearly two-thirds of the days in the study. Regarding weigh-ins, the Lark coach encourages participants to weigh themselves at least once per week. On average, participants completed a weigh-in on 24.3 (SD 19.1; median 18.0; 95% CI 22.6-26.0) days, or approximately 2 times per week over the course of the study. Of these weigh-ins, participants provided the vast majority (435/489, 88.9%) by stepping on their cellular scale, with only 11% (54/489) self-reported within the app. Participants completed an average of 7.1 (SD 4.1; median 9.0; 95% CI 6.7-7.5) educational lessons during the 90-day study.

### Primary Objective 3: Screener Submission

For objective 3, we found that participants were highly engaged with the screeners in the app. Specifically, 89.8% (440/489; 95% CI 87%-92.5%) submitted all 3 screeners (*excellent*), 94.3% (460/489; 95% CI 91.6%-96%) submitted ≥2 screeners (*good*), and 97.5% (479/489; 95% CI 96.3%-99%) submitted ≥1 screeners (*minimum improvement over usual care*). It should be noted that these groups were not mutually exclusive.

### Secondary Objective: Weight Loss

We examined peak weight loss among participants who provided at least 2 weights (483/489, 98.8%). Across this sample, the average weight nadir was 3.8% (SD 2.9%; 95% CI 3.5%-4.1%) and occurred on day 51.9 (SD 26.4; 95% CI 49.5-54.3). There was a significant correlation between the day in the study that the weight nadir occurred and percentage weight loss at nadir date (*r*=0.36; *P*<.001). This indicates a higher percentage weight loss for weight nadir dates later in the study. Across all participants, the vast majority (482/483, 99.9%) lost or maintained weight, with only 1 (0.2%) of the 483 members gaining weight. The proportion of the sample in 4 different weight loss categories is as follows: 26.5% (128/483) lost weight ≥5%, 44.7% (216/483) lost weight between 2% and 4.9%, 28.6% (138/483) maintained weight –2% to +2%, and 0.2% (1/483) gained weight >2% [39,40]. We also examined weight loss by baseline BMI and found that peak weight loss was not significantly correlated with BMI (*r*=0.004; *P*=.41).

## Discussion

### Summary

The study results indicate that the fully digital AI-powered health coaching app called Lark Heart Health had a high degree of acceptability and feasibility among this pilot study sample. In this 90-day study of the Heart Health program, we found that participants were highly satisfied with their experience using the app, showed a high degree of engagement with health coaching and other app features, and successfully completed screeners on important health indicators during their first month in the program. Secondary analyses further indicated that participants lost weight during the 90-day program. Taken together, the findings from this pilot study met or exceeded all success criteria for the primary study objectives and provided key insights into weight loss as a clinical outcome that can be further examined in future clinical studies. Thus, these findings support the acceptability and feasibility of the Heart Health program. We discuss key insights from each of the primary objectives in the next subsection.

### Insights From Primary Objectives

Regarding objective 1, the 2 participant satisfaction surveys indicated that participants were highly satisfied with their experience using the Heart Health program, including their experience taking the ASCVD Risk Estimator survey in the app. Mean scores on all items across the 2 surveys indicated that participants rated their experience favorably across multiple dimensions, such as how easy it was to understand the information in the app and feeling comfortable with taking a survey in the app. The mean score on the overall app experience survey (4.4 out of 5; SD 0.8) also closely aligns with *high satisfaction* scores in prior feasibility studies on apps for CVD management [24].

For objective 2, we found that participants were highly engaged with coaching in the Heart Health app. In month 1, of the 489 participants, 483 (98.8%) met the success criteria for being highly engaged (ie, ≥5 coaching conversations); in month 2, there were 445 (91%) highly engaged participants; and in month 3, there were 408 (83.4%) highly engaged participants. Engagement with the app far exceeded the expectations set by our success criteria. This insight indicates that engagement thresholds can be set at higher levels in future testing with the Heart Health program. Although monthly engagement rates were higher than anticipated across all 3 months, the results showed a decrease in engagement over time; for instance, 8.6% (42/489) of the participants were *minimally engaged* in month 3 (the final month in the study), as indicated by ≤1 coaching conversation during this month. This decrease is expected in digital health, with engagement and retention rates typically dropping over time [41,42]. Identifying this decrease in engagement also provides the opportunity to identify when engagement begins to wane and perhaps use targeted incentives at this time to mitigate drop-off. As we focused on coaching conversations as the primary engagement outcome, it is also possible that some users may have favored different features, such as weight logging and device use (eg, cellular scale). The descriptive results on weigh-ins support the assertion that users engaged with this feature.

The results from objective 2 also highlight the high degree of variability in engagement between participants. As with any program or app, different individuals will have different use patterns or connect with different aspects of the program. Indeed, we have shown in previous research of other Lark programs that there are different user *personas* among individuals who engage with the app, with some users focusing more on data and tracking functionalities or device use, rather than being highly engaged with coaching specifically [43].

In the context of this pilot study, there were some participants who had disproportionately high engagement levels. In our previous work, we refer to these individuals as *enthusiasts* [43]. These are individuals who log on many times per day and engage with many or all facets of the app. It is important to note that super users are not representative of most users of an app and can skew mean scores; for example, the median values for coaching conversations in this study were lower than the average values, although this still indicated high engagement. That said, designers of apps can gain significant insights for program improvement from qualitative interviews with both high engagers and low engagers alike [41].

Regarding objective 3, the results showed high participant submission rates for screeners focused on important health indicators, including ASCVD risk, medication adherence, and depressive symptoms. Specifically, 90% (440/489) of the participants achieved the a priori benchmark of submitting all 3 health screeners. This result indicates that participants were willing and able to complete these screeners using the app. This is a key result for the feasibility and acceptability of taking screeners in the Heart Health app because prior work has highlighted that it is important for patients in heart health prevention to show high levels of approval for risk surveys [34]. Moreover, these findings support the use of the Heart Health app as a user-friendly solution that enables patients to complete important health screeners outside of the primary care setting. This is an important use because there are significant barriers to screener completion during patient visits, such as short appointment times, workflow disruption, and a lack of patient preparedness [19,20,27]. Moreover, screener completion has been shown to have several benefits to patients; for instance, health screeners can help increase patient awareness and knowledge of their values on key CVD risk factors (eg, cholesterol level and blood pressure) [20].

The acceptability of screener submission in the app also has important implications for future delivery of the Heart Health program. In a commercial version of the program, participants would complete health screeners, such as the PHQ-2, MAQ, and ASCVD Risk Estimator, every 60 to 90 days. Longitudinal self-monitoring has many benefits for the self-management of CVD, such as enabling participants to track changes in their symptoms over time [44] and improve medication adherence [45]. Further integration of the Heart Health app with health partners would also enable participants’ health providers to track their patients’ changes over time and potentially support clinical decision-making [20,46].

### Weight Loss and Study Retention

Beyond the primary acceptability and feasibility study results, secondary analyses also revealed initial insights into weight loss during the program. Specifically, participants showed an average peak weight loss of 3.8% (SD 2.9%) during the study, and weight loss percentage was not correlated with baseline BMI. This percentage weight loss compares favorably to that in prior studies examining weight loss in digital health interventions [47]. These results should be considered preliminary because the pilot study was not developed with the intention of testing weight loss or other clinical outcomes. However, these results support the future consideration of weight loss as a clinical outcome for the Heart Health program.

We observed an 80% (407/509) retention rate in month 3 of the study. This retention rate is on par with target benchmarks from non-AI digital health studies that are higher touch than Heart Health [38]. In addition, the withdrawal rate for the study was low: only 20 (3.9%) of the 509 participants withdrew after enrollment. Withdrawal rates for digital health apps with frequent survey content range from 15% to 51% [48]. It is important to note that the retention rates reported here should be interpreted as *research study* retention rates; study retention rates are typically higher than retention rates in programs that are commercially released to real-world populations. Indeed, it is well established that retention is one of the biggest challenges with digital health solutions, and there are often high attrition rates with health programs delivered via mobile apps [41,42]. Improving retention rates in real-world populations is a major focus in the digital health space [49] and will be a focal point in a commercial version of the Heart Health program.

### Limitations and Future Directions

There are several limitations to the study results. First, this was a pilot study examining feasibility and acceptability as a first step in the evidence-generation process; the findings presented here are not meant to show the clinical efficacy of the Heart Health program and should not be interpreted as such. Future studies could examine the impact of the Heart Health program in a controlled trial; for example, a key next step in evidence generation is to examine clinical outcomes (eg, weight loss) between program participants and a control group receiving usual care or a control group that involves human coaching. As a second limitation, the sample used for this study contained individuals recruited to participate in a research study rather than real-world participants of a commercial version of the product. As such, an important future direction for generating evidence for the Heart Health program will be examining these objectives and other study outcomes in a commercial version of the product with a real-world sample. In addition, this study included opportunities for incentives throughout the Heart Health program that are similar to incentives provided in existing Lark programs but may not be identical to incentives provided in the commercial implementation of the Heart Health program. Importantly, only 1 incentive was directly tied to any type of app engagement: the Fitbit device incentive provided for completing the 90-day program. It is common for research studies to provide an incentive for study completion, and it is also common for Lark’s existing health care partners to provide members with a Fitbit device for reaching a major program milestone. As a result, we do not believe that this incentive had any undue impact on participant engagement that would differ significantly from the commercial implementation of the program. Differences in incentive structure may have an impact on participant engagement in future program implementation, and this will be dependent on health care partners.

A third limitation is that the sample in this study came primarily from a single health partner in California, and participants tended to be older White adults from this region. Future work should examine the impact of the program among diverse samples; indeed, this is a key limitation and a future direction in the broader digital health space [12,42]. Finally, a key next step in improving the program is to gain a deeper understanding of the user experience with the app; this could include qualitative or interview-based studies with individuals who show different engagement patterns (eg, low engagers, average engagers, and super users) to gather detailed feedback on features that could be improved.

### Conclusions

These acceptability and feasibility results provide a key first step in the process of evidence generation for a new AI-powered digital program for heart health. These results indicate that participants in this study were satisfied with their experience using the Heart Health app, highly engaged with app features, and willing and able to complete health screening surveys in the app. While digital health solutions are increasingly common, many have not been tested using formalized evidence-generation strategies that are traditionally used for new therapeutics, such as pharmaceuticals [13]. As such, the results from this pilot study provide an example blueprint for early-stage evidence generation for new digital health offerings in the prevention and management of CVD.

## Appendix

Multimedia Appendix 1 Additional details on the program, study design, and participant characteristics measurement.

## Abbreviations

ACC: American College of Cardiology
AHA: American Heart Association
AI: artificial intelligence
ASCVD: atherosclerotic cardiovascular disease
CRO: contract research organization
CVD: cardiovascular disease
HIPAA: Health Insurance Portability and Accountability Act
HITRUST: Health Information Trust Alliance
MAQ: Medication Adherence Questionnaire
PHQ-2: Patient Health Questionnaire-2
SOC 2: Service Organization Control 2

## Data Availability

The data set analyzed during this study is not publicly available due to the fact that these analyses include sensitive, potentially identifiable information that was obtained through a collaboration between Lark Health and its health care partners but data may be available from the corresponding author on reasonable request.
