# Supplementary material for: Evaluating a New Digital App–Based Program for Heart Health: Feasibility and Acceptability Pilot Study
Source: JMIR Form Res. 2024 May 24;8:e50446. doi: 10.2196/50446 (PMC11161712; doi:10.2196/50446)
Supplement: Multimedia Appendix 1 [file formative_v8i1e50446_app1.docx]

**Supplemental Materials**

**Additional Details on the Heart Health Program and Study Design**

Enrolled participants completed a 90-day active participation phase. Participation activities involved the participant’s interaction with the AI coach in interactions referred to as “coaching conversations.” The AI coach and participants conversed via a digital, text-message-based interface that provides personalized educational material about CVD and sustainable methods to make practical health-promoting behavior changes. As described in detail in previous publications, Lark coaching uses elements of behavior change theories and techniques including positive psychology, cognitive behavioral therapy, and motivational interviewing to help participants make sustainable lifestyle changes to improve their health (Graham et al., 2022).

A key component of the Heart Health coaching conversations is the delivery of educational lessons. The AI coach delivers each educational lesson over the course of a week, with content spread out over 7 “check-ins.” Heart Health study participants could complete up to 12 educational lessons during the pilot study. Examples of educational lesson topics include: *Risk Factors for Heart Disease, Activity for Low Blood Pressure, Understanding Cholesterol, Eat Well,* and *Find Time for Fitness.* Coaching conversations also included prompts for meal and physical activity logging. This enabled the digital coach to engage users with content and feedback specifically related to their manually logged data and connected-device weight readings from the cellular-connected scale.

Additional study activities focused on completing surveys, including the following health screening surveys in the app: An ASCVD risk estimator survey, a medication adherence questionnaire, and a depression screener (described in further detail in the *Measures* section). According to the participant’s preferences and coaching interactions, they were also presented with additional surveys about medication adherence, smoking habits, self-efficacy, and other topics. Lastly, the study included two phone calls that occurred at the beginning of Month 2 and Month 3. Personnel from Lark Health’s contract research organization (CRO) partner scheduled these calls with each participant and provided an opportunity for participants to provide feedback in the form of two satisfaction surveys.

**Participant Characteristics Measure Description**

We assessed participant characteristics for the study sample using a modified version of the “Non-Laboratory” Based INTERHEART Modifiable Risk Score survey (McGorrian et al., 2011; Yusuf et al., 2014). This brief self-report questionnaire includes assessment of age, sex, height and weight (for calculation of BMI), history of high blood pressure, history of diabetes, history of tobacco use and exposure, depression in the past year, physical activity during leisure time, and typical dietary habits. The instrument classifies typical physical activity in leisure time as mainly sedentary, mild exercise with minimal effort, moderate exercise, or strenuous exercise. The instrument classifies typical dietary habits by assessing responses to five unhealthy diet items: 1) eating salty food daily, 2) eating deep fried foods 3x/week or more, and 3) eating meat 2x/day or more, 4) not eating fruit daily and 5) not eating vegetables daily.
